# NUTRITIONAL STATUS, PHYSICAL ACTIVITY, SEDENTARY BEHAVIOR, DIET, AND LIFESTYLE IN CHILDHOOD: AN ANALYSIS OF RESPIRATORY DISEASES IN ADOLESCENCE

**DOI:** 10.1590/1984-0462/2021/39/2020007

**Published:** 2020-11-27

**Authors:** Eduardo Rossato de Victo, Gerson Ferrari, Victor Keihan Rodrigues Matsudo, Carlos André Miranda Pires, Timóteo Leandro Araújo, Peter Todd Katzmarzyk, Dirceu Solé

**Affiliations:** a Discipline of Allergy, Clinical Immunology and Rheumatology, Department of Pediatrics, Universidade Federal de São Paulo (UNIFESP), São Paulo, SP, Brazil.; b Laboratory of Sciences of Physical Activity, Sports and Health, Faculty of Medical Sciences, Universidad de Santiago de Chile, Santiago, Chile.; c Centro de Estudos do Laboratório de Aptidão Física de São Caetano do Sul, São Caetano do Sul, SP, Brazil.; d Center for research in Neuropsychology and Cognitive and Behavioral Intervention, Faculty of Psychology and Educational Sciences, Universidade de Coimbra, Portugal.; e Pennington Biomedical Research Center, Baton Rouge, LA, United States of America.

**Keywords:** Asthma, Rhinitis, Lifestyle, Diet, Motor activity, Asma, Rinite, Estilo de vida, Dieta, Atividade motora

## Abstract

**Objective:**

To evaluate the association between nutritional status, physical activity, sedentary behavior, diet, and lifestyle in childhood with respiratory diseases during adolescence.

**Methods:**

Prospective study conducted in São Caetano do Sul, São Paulo - Brazil, as part of the *International Study of Childhood Obesity, Lifestyle and the Environment* (ISCOLE). During childhood, indicators of lifestyle (body composition, physical activity, sedentary behavior, diet) and family environment were measured in 2012 and 2013. After five years, participants answered the *International Study of Asthma and Allergies in Childhood* (ISAAC) questionnaire for the diagnosis of respiratory diseases (asthma and/or rhinitis). Analyses were determined by logistic regression.

**Results:**

A total of 168 schoolchildren (56% boys) were evaluated, and the prevalence of asthma and rhinitis accounted for 15.5 and 25.6%, respectively. Whole milk consumption (Odds Ratio [OR]=1.24; 95% confidence interval [95%CI] 1.03-1.49), having a television in the bedroom (OR=0.29; 95%CI 0.12-0.71), and attending physical education classes ≥2 times/week (OR=0.30; 95%CI 0.11-0.81) in childhood were associated with the presence of asthma in adolescence. Factors significantly associated with rhinitis were as follows: female participants (OR=2.45; 95%CI 1.20-4.98) and whole milk consumption (OR=1.21; 95%CI 1.04-1.40).

**Conclusions:**

Higher consumption of whole milk, not having a television in the bedroom, few physical education classes, and being a girl were factors associated with respiratory diseases. Public policies should be directed toward a healthier lifestyle and the prevention of respiratory diseases.

## RESUMO

**Objetivo:**

Avaliar a associação entre estado nutricional, atividade física, comportamento sedentário, dieta e estilo de vida na infância e doenças respiratórias durante a adolescência.

**Métodos:**

Estudo prospectivo realizado em São Caetano do Sul, São Paulo, como parte do *International Study of Childhood Obesity, Lifestyle and the Environment*. Indicadores do estilo de vida (composição corporal, atividade física, comportamento sedentário, dieta) e do ambiente familiar foram mensurados durante a infância, em 2012 e 2013. Após cinco anos, os escolares responderam o questionário do *International Study of Asthma and Allergies in Childhood* (ISAAC) para o diagnóstico de doenças respiratórias (asma e/ou rinite). Análises foram determinadas por meio da regressão logística.

**Resultados:**

Foram avaliados 168 escolares (56% meninos), e as prevalências de asma e rinite observadas foram 15,5 e 25,6%, respectivamente. Consumir leite integral (*Odds Ratio* [OR]=1,24; intervalo de confiança 95% [IC95%] 1,03-1,49), ter televisão no quarto (OR=0,29; IC95% 0,12-0,71) e praticar aulas de educação física ≥2 vezes/semana (OR=0,30; IC95% 0,11-0,81) na infância foram associados com a presença de asma na adolescência. Os fatores significativamente associados à rinite foram: ser do sexo feminino (OR=2,45; IC95% 1,20-4,98) e consumir leite integral (OR=1,21; IC95% 1,04-1,40).

**Conclusões:**

Consumir mais leite integral, não ter televisão no quarto, ter tido poucas aulas de educação física e ser do sexo feminino foram fatores associados às doenças respiratórias. Políticas públicas devem ser dirigidas para um estilo de vida mais saudável e a prevenção de doenças respiratórias.

## INTRODUCTION

Asthma and rhinitis are chronic respiratory diseases with high prevalence, compromising the quality of life of the affected population and, consequently, causing public health problems.^1^ Such diseases are associated with children’s inappropriate habits, such as reduced physical activity (PA) and increased time of sedentary behavior (SB), thus favoring overweight and childhood obesity.^2^ ^,^ ^3^

According to data from the National Scholar Health Survey (*Pesquisa Nacional de Saúde do Escolar* - PeNSE), Brazilian adolescents account for the highest worldwide prevalence of asthma (23.2%).^4^ Conversely, in children, the prevalence of asthma and allergic rhinitis is 24.3 and 12.6%, respectively.^5^ Over a nine-year period (2003-2012), the prevalence of asthma stabilized (18.5 and 17.5%) and allergic rhinitis increased from 16.2 to 20.6% among adolescents.^6^

In addition to the genetic aspect, factors, such as parental smoking, family history, lifestyle, environment, and social conditions, are associated with the occurrence of asthma and allergic rhinitis.^7^ ^,^ ^8^ A multicenter study on children from different regions of Brazil evaluated risk factors associated with asthma and found that the identified factors were not universal among the 11 centers. For example, lack of vegetable consumption was associated with asthma in 4/11 of the centers, and the performance of PA <2 times/week was identified as a risk factor for asthma in 2/6 of the evaluated groups.^7^

Moreover, some aspects in childhood can increase the risk of developing asthma and rhinitis in adolescence and even in adulthood such as poor nutritional status and physical inactivity.^9^ ^,^ ^10^ A prospective study found a positive association between body mass index (BMI) in childhood and the rate of hospitalization due to asthma in adulthood and the association, among women, of high BMI in childhood and the highest risk of hospitalization due to asthma in adulthood when compared with those with normal or low BMI in childhood.^9^ On the other hand, among men, low BMI in childhood increased the risk of asthma in adulthood compared with those with normal or high BMI.^10^ In addition to BMI, childhood physical inactivity was also positively associated with the development of asthma and rhinitis in late childhood.^10^ Such studies were carried out in high-income countries, and these studies are scarce in low- and middle-income countries such as Brazil. Furthermore, objective instruments, such as accelerometers, have greater sensitivities to assess different PA intensities than questionnaires.^11^ Thus, the present study aimed to assess the association between nutritional status, PA, SB, diet, and lifestyle in childhood and respiratory diseases during adolescence.

## METHOD

Prospective study characterized by two phases: in the first phase, the sample population was in childhood (9-11 years old), and in the second, in adolescence (14-16 years old). The first phase consisted of Brazilian data from the *International Study of Childhood Obesity, Lifestyle and the Environment* (ISCOLE). ISCOLE is a multicenter study carried out in 12 countries, with data collection carried out in 2012 and 2013, including data on lifestyle, diet, PA, and family environment, following a rigorous training and certification system.^12^ In total, 500 students (aged 9-11 years), regularly enrolled in public and private schools in the municipality of São Caetano do Sul, state of São Paulo, Brazil, were included. The municipality has an estimated population of 159,608 inhabitants and has one of the best Human Development Indexes in Brazil (0.86).^13^ ^,^ ^14^ In order for the sample to be homogeneous in terms of socioeconomic level, the proportion of four public schools (low and lower middle class) to one private school (middle and upper middle class) was adopted. Details from ISCOLE Brazil on recruiting students and collecting data have been previously published.^15^

The second phase took place in 2018, when the sample population was already in adolescence, with the application of a telephone survey to assess the presence or absence of asthma and rhinitis.^16^ According to the sample calculation, based on the estimation of a prevalence of 20.6% (value estimated for the prevalence of rhinitis),^6^ associated with a 95% confidence interval (95%CI) and a maximum sampling error of 5%, 168 schoolchildren were required. The sample was selected by a simple random drawing, using a numbered list of students. In the absence of the selected individual or the refusal to participate in the study, the next subject was selected. This criterion was used until reaching the required number of students, according to the sample calculation. Parents and/or guardians signed the informed consent form, and the students signed the consent form, according to Resolution No. 196/96 of the National Health Council of Brazil. The ISCOLE study protocol was approved by the Research Ethics Committee of Universidade Federal de São Paulo.

Height (cm) was assessed using the Seca 213 portable stadiometer (Seca^®^, Hamburg, Germany) with students barefoot and with their head positioned in the Frankfurt plane. Body weight (kg) and body fat percentage (%BF) were obtained using the Tanita SC-240 bioelectrical impedance scale (Arlington Heights, Illinois, United States of America), with students barefoot and wearing minimal clothing. BMI (kg/m^2^) was calculated and classified according to the Z-score, based on the reference growth curves proposed by the World Health Organization (WHO),^17^ being classified as: underweight: <−2 standard deviations (SD); normal weight: −2 SD to 1 SD; overweight: >1 DP to 2 DP; obesity: >2 SD.^17^

Waist circumference was measured with a non-elastic and metallic anthropometric tape, with the skin exposed and after normal expiration (located between the margin of the lower rib and the iliac crest), with the students in an upright body position, with their feet together and arms relaxed.^12^

To objectively monitor PA and SB, ActiGraph GT3X accelerometers (ActiGraph^®^, United States of America) were used. The device was placed on the right side of the body, on the midaxillary line, and attached to an elastic waistband. Moderate-to-vigorous physical activity (MVPA) and SB were obtained after encouraging students to use the accelerometer 24 hours/day, for at least seven days (including two weekend days). The minimum accepted amount of data was four days (with at least 10 hours/day and including one day at the weekend, disregarding the sleeping hours).^18^ Data were collected for analysis at a sampling rate of 80 Hz, downloaded in periods of one second, and aggregated for periods of 15 seconds.^19^

PA was categorized according to the quantity of counts (unit of measurement of accelerometers) for periods of 15 seconds as follows: sedentary time: ≤25 counts; light physical activity (LPA): >25 to 573 counts ; moderate physical activity (MPA): ≥574 to 1,002 counts; vigorous physical activity (VPA): ≥1,003 counts; moderate-to-vigorous physical activity (MVPA): ≥574 counts*.* ^19^

Diet was evaluated using the *Diet and Lifestyle Questionnaire*, which comprises questions about the weekly food frequency through a list composed of 23 foods and beverages.^12^ The main components were analyzed to identify the students’ eating patterns. With the scores obtained in these analyses, diet was classified as: healthy (vegetables, orange, fruit juices, fruits, among others) and unhealthy (fast food, french fries, ice creams, sweets, pies, among others).^20^

The same questionnaire comprises questions about screen time (television, video game, and computer) of students on weekdays and weekends.^20^ Answers regarding the time they slept and woke up were also obtained, and the total sleep time was subsequently calculated.^20^ Sleep time was classified as adequate when following the recommendation of 9-12 hours/day of sleep, and inadequate, when it did not follow this recommendation.^21^

Parents and/or legal guardians were asked to complete the *Home Environment Questionnaire* ^12^ and the *Demographic and Family Health Questionnaire.* ^12^ Information was obtained on the annual family income, in the Brazilian currency (*reais*, R$), the parents’ level of education, in addition to questions about the child’s and family’s health history as well as the parents’ body weight and height. All details of the questionnaire have been previously provided.^12^

The students answered the *International Study of Asthma and Allergies in Childhood* (ISAAC) questionnaire by telephone interview in order to assess the presence or absence of asthma and/or rhinitis. Initially, the questionnaire was validated for Brazilian children^22^ and later for the application via telephone.^16^

Participants who positively answered to the question “Have you had wheezing in the past 12 months?” were classified as having asthma. To be classified as having rhinitis, the answer to the question “In the past 12 months, have you had any problems with sneezing, runny nose, or nasal obstruction without having the flu or a cold?” should be positive.^23^

Telephone interviews for the application of the ISAAC questionnaire took place in the 2^nd^ period of the study, in 2018. Answers were provided after the explanation and authorization of the parents and/or guardians. Furthermore, the questionnaire was applied in the presence of the parents and/or guardians and, when possible, using the speakerphone, following the validated protocol^16^ for applying the questionnaire via telephone.

The Kolmogorov-Smirnov test was used to assess the normality of the data. According to the nature of the variables, they were presented as mean and standard deviation or frequency and percentage. The reliability of the healthy and unhealthy eating scales of the *Diet and Lifestyle Questionnaire* was assessed and validated by the Cronbach’s alpha coefficient, being classified as acceptable for presenting values above 0.75.

All analyses were performed using the IBM *Statistical Package for the Social Sciences* (SPSS) Statistics for Windows software, version 22 (IBM, 2013). Depending on the studied variables, parametric (Student’s *t* or ANOVA) and non-parametric (Chi-square or Fisher’s exact) tests were applied, considering the level of rejection for the null hypothesis at 5%. Stepwise forward logistic regression was carried out to identify factors associated with asthma and rhinitis. Only variables with p<0.20 were included in the model. The existence of possible multicollinearity between the independent variables was verified using the variance inflation factor (acceptable: <10).

## RESULTS

The sample included 168 students, and 56% of them were boys. Of the total sample, 26 students (15.5%) were classified as having asthma and 43 (25.6%), rhinitis. In Tables 1 to 4 the different characteristics and behaviors of students during childhood (9-11 years old) are presented, according to the presence or absence of asthma and rhinitis during adolescence. Categorical BMI, whole milk consumption, having a television in the bedroom, and attending physical education classes showed significant differences between those who were classified with and without asthma during adolescence (Tables 1 and 2). Only sex and whole milk consumption showed significant differences between those who were classified with and without rhinitis, as demonstrated in Tables 3 and 4.

**Table 1:** Description [mean(standard deviation); n(%)] of anthropometric variables and those related to physical activity of groups with and without asthma.

	Total (n=168)	Without asthma (n=142)	With asthma (n=26)	p-value
Age (years) (n=168)	10.4 (0.4)	10.4 (0.4)	10.38 (0.5)	0.458*
Sex (n=168)				
Boys	94 (55.0%)	80 (47.6%)	14 (8.4%)	0.814**
Girls	74 (45.0%)	62 (36.9%)	12 (7.1%)	
Ethnicity (n=167)				
White	130 (77.8%)	110 (65.9%)	20 (12%)	0.786**
Black	14 (8.4%)	11 (6.6%)	3 (1.8%)	
Other	23 (13.8%)	20 (12%)	3 (1.8%)	
Height (cm) (n=168)	144.23 (7.6)	144.47 (7.6)	142.93 (7.79)	0.348*
Body weight (kg) (n=168)	41.12 (11.9)	41.08 (10.9)	41.35 (17.08)	0.918*
Body fat (%) (n=162)	22.43 (9.4)	22.38 (8.6)	22.69 (12.98)	0.878*
BMI (kg/m^2^) (n=168)	19.52 (4.4)	19.47 (3.9)	19.76 (6.31)	0.760*
Categorical BMI (n=168)				
Underweight	4 (2.4%)	2 (1.2%)	2 (1.2%)	0.040**
Normal weight	86 (51.2%)	72 (42.9%)	14 (8.3%)	
Overweight	33 (19.6%)	32 (19%)	1 (0.6%)	
Obese	45 (26.8%)	36 (21.4%)	9 (5.4%)	
Waist circumference (cm) (n=168)	66.8 (10.9)	66.9 (10.3)	66.0 (14.4)	0.685*
Total sedentary time (min/day) (n=155)	496.8 (64.9)	495.3 (67.4)	505.7 (51.1)	0.445*
LPA (min/day) (n=155)	339.9 (50.4)	342.3 (50.4)	328.6 (50.0)	0.207*
MPA (min/day) (n=155)	42.7 (15.9)	43.3 (16.2)	39.7 (14.3)	0.304*
MVPA (min/day) (n=155)	62.0 (27.1)	62.9 (27.8)	57.5 (23.3)	0.350*
VPA (min/day) (n=155)	19.4 (12.7)	19.7 (13.1)	17.7 (10.7)	0.478*
MVPA recommendations (n=155)				
Followed the recommendations	73 (47.1%)	60 (38.7%)	13 (8.4%)	0.745**
Did not follow the recommendations	82 (52.9%)	69 (44.5%)	13 (8.4%)	

Values presented as means (standard deviation) for the quantitative variables and n(%) for the qualitative variables; BMI: body mass index; LPA: light physical activity; MPA: moderate physical activity; MVPA: moderate-to-vigorous physical activity; VPA: vigorous physical activity.

*p-value of the Student’s *t-*test; **p-value of the Chi-square test.

**Table 2:** Description [mean(standard deviation); n(%)] of the variables of diet, lifestyle, socioeconomic level, parents’ level of education, and parents’ body mass index of groups with and without asthma.

	Total	Without asthma	With asthma	p-value
Healthy diet (n=167)	2.98 (0.85)	2.95 (0.83)	3.13 (0.92)	0.328*
Unhealthy diet (n=167)	3.99 (1.23)	3.94 (1.21)	4.27 (1.30)	0.210*
Fruit consumption (n=167)	4.63 (1.71)	4.55 (1.73)	5.08 (1.55)	0.147*
Vegetable consumption (n=167)	4.11 (1.92)	4.04 (1.94)	4.50 (1.77)	0.265*
Skimmed milk consumption (n=167)	3.60 (2.44)	3.54 (2.41)	3.96 (2.60)	0.418*
Whole milk consumption (n=167)	3.85 (2.44)	3.66 (2.40)	4.88 (2.39)	0.018*
Fast food consumption (n=167)	2.98 (1.49)	2.96 (1.53)	3.12 (1.27)	0.622*
Soft drink consumption (n=167)	3.74 (1.75)	3.72 (1.72)	3.88 (1.92)	0.654*
Screen time (hours/day) (n=167)	3.90 (2.29)	3.91 (2.31)	3.86 (2.25)	0.920*
Screen time (n=167)				
<2 hours/day	46 (27.5%)	39 (23.4%)	7 (4.2%)	0.938**
>2 hours/day	121 (72.5%)	102 (61.1%)	19 (11.4%)	
Sleep time (n=167)				
Adequate (%)	73 (43.7%)	63 (37.7%)	10 (6.0%)	0.557**
Inadequate	94 (56.3%)	78 (46.7%)	16 (9.6%)	
Transportation to school (n=167)				
Active	64 (38.3%)	57 (34.1%)	7 (4.2%)	0.193**
Passive	103 (61.7%)	84 (50.3%)	19 (11.4%)	
Television in the bedroom (n=165)				
Yes	123 (74.5%)	110 (66.7%)	13 (7.9%)	0.005**
No	42 (25.5%)	30 (18.2%)	12 (7.3%)	
Physical education classes (n=167)				
<2 classes/week	25 (15%)	17 (10.2%)	8 (4.8%)	0.014**
≥2 classes/week	142 (85%)	124 (74.3%)	18 (10.8%)	
Family income (n=153)				
<BRL 19,620.00	49 (32%)	41 (26.8%)	8 (5.2%)	0.537**
BRL 19,621.00 to BRL 32,700.00	36 (23.5%)	30 (19.6%)	6 (3.9%)	
BRL 32,701.00 to BRL 58,860.00	36 (23.5%)	30 (19.6%)	6 (3.9%)	
>BRL 58,860.00	32 (20.9%)	30 (19.6%)	2 (1.3%)	
Parents’ combined level of education (n=167)				
Some High School	27 (16.2%)	23 (13.8%)	4 (2.4%)	0.989**
High School	84 (50.3%)	71 (42.5%)	13 (7.8%)	
Higher Education	56 (33.5%)	47 (28.1%)	9 (5.4%)	
Mother’s BMI (n=160)	26.05 (4.96)	25.96 (5.05)	26.58 (4.48)	0.562*
Father’s BMI (n=141)	27.08 (3.70)	27.12 (3.93)	26.89 (2.04)	0.801*

Values presented as means (standard deviation) for the quantitative variables and n(%) for the qualitative variables; BMI: body mass index.

*p-value of the Student’s *t-*test; **p-value of the Chi-square test; BRL: Brazilian currency.

**Table 3:** Description [mean(standard deviation); n(%)] of anthropometric variables and those related to physical activity of groups with and without rhinitis.

	Total (n=168)	Without rhinitis (n=125)	With rhinitis (n=43)	p-value
Age (years) (n=168)	10.4 (0.4)	10.5 (0.4)	10.4 (0.4)	0.556*
Sex (n=168)				
Boys	94 (56%)	77 (45.8%)	17 (10.1%)	0.012**
Girls	74 (46%)	48 (28.6%)	26 (15.5%)	
Ethnicity (n=167)				
White	130 (77.8%)	94 (56.3%)	36 (21.6%)	0.540**
Black	14 (8.4%)	11 (6.6%)	3 (1.8%)	
Other	23 (13.8%)	19 (11.4%)	4 (2.4%)	
Height (cm) (n=168)	144.2 (7.6)	144.2 (7.7)	144.4 (7.6)	0.907*
Body weight (kg) (n=168)	41.1 (11.9)	40.9 (11.2)	41.8 (14.0)	0.654*
Body fat (%) (n=162)	22.4 (9.4)	21.9 (8.7)	23.6 (11.1)	0.334*
BMI (kg/m^2^) (n=168)	19.5 (4.4)	19.4 (4.1)	19.9 (5.3)	0.656*
Categorical BMI (n=168)				
Underweight	4 (2.4%)	3 (1.8%)	1 (0.6%)	0.996**
Normal weight	86 (51.2%)	64 (38.1%)	22 (13.1%)	
Overweight	33 (19.6%)	25 (14.9%)	8 (4.8%)	
Obese	45 (26.8%)	33 (19.6%)	12 (7.1%)	
Waist circumference (cm) (n=168)	66.8 (10.96)	66.5 (10.0)	67.6 (13.5)	0.574*
Total sedentary time (min) (n=155)	496.8 (64.91)	493.3 (66.2)	508.6 (59.8)	0.217*
LPA (min/day) (n=155)	339.9 (50.42)	339.3 (49.9)	342.4 (52.8)	0.747*
MPA (min/day) (n=155)	42.7 (15.90)	43.7 (15.5)	39.3 (16.9)	0.147*
MVPA (min/day) (n=155)	62.0 (27.13)	63.9 (26.9)	55.6 (27.1)	0.107*
VPA (min/day) (n=155)	19.4 (12.72)	20.3 (12.9)	16.3 (11.6)	0.106*
MVPA recommendations (n=155)				
Followed the recommendations	73 (47.1%)	61 (39.4%)	21 (13.5%)	0.456**
Did not follow the recommendations	82 (52.9%)	58 (37.4%)	15 (9.7%)	

Values presented as means (standard deviation) for the quantitative variables and n(%) for the qualitative variables; BMI: body mass index; LPA: light physical activity; MPA: moderate physical activity; MVPA: moderate-to-vigorous physical activity; VPA: vigorous physical activity.

*p-value of the Student’s *t-*test; **p-value of the Chi-square test.

**Table 4:** Description [mean(standard deviation); n(%)] of the variables of diet, lifestyle, socioeconomic level, parents’ level of education, and parents’ body mass index of groups with and without rhinitis.

	Total	Without rhinitis	With rhinitis	p-value
Healthy diet (n=167)	2.98 (0.85)	2.95 (0.80)	3.06 (0.97)	0.461*
Unhealthy diet (n=167)	3.99 (1.23)	3.99 (1.24)	3.99 (1.19)	0.970*
Fruit consumption (n=167)	4.63 (1.71)	4.61 (1.70)	4.67 (1.77)	0.840*
Vegetable consumption (n=167)	4.11 (1.92)	4.06 (1.96)	4.28 (1.80)	0.514*
Skimmed milk consumption (n=167)	3.60 (2.44)	3.62 (2.49)	3.56 (2.29)	0.885*
Whole milk consumption (n=167)	3.85 (2.44)	3.56 (2.40)	4.67 (2.38)	0.010*
Fast food consumption (n=167)	2.98 (1.49)	2.87 (1.41)	3.30 (1.70)	0.103*
Soft drink consumption (n=167)	3.74 (1.75)	3.78 (1.71)	3.63 (1.88)	0.620*
Screen time (hours/day) (n=167)	3.90 (2.29)	3.87 (2.25)	3.99 (2.44)	0.772*
Screen time (n=167)				
<2 hours/day	46 (27.5%)	32 (19.2%)	14 (8.4%)	0.393**
>2 hours/day	121 (72.5%)	92 (55.1%)	29 (17.4%)	
Sleep time (n=167)				
Adequate (%)	73 (43.7%)	56 (33.5%)	17 (10.2%)	0.522**
Inadequate	94 (56.3%)	68 (54.8%)	26 (15.6%)	
Transportation to school (n=167)				
Active	64 (38.3%)	52 (31.1%)	12 (7.2%)	0.103**
Passive	103 (61.7%)	72 (43.1%)	31 (18.6%)	
Television in the bedroom (n=165)				
Yes	123 (74.5%)	93 (56.4%)	30 (18.2%)	0.591**
No	42 (25.5%)	30 (18.2%)	12 (7.3%)	
Physical education classes (n=167)				
<2 classes/week	25 (15%)	16 (9.6%)	9 (5.4%)	0.204**
≥2 classes/week	142 (85%)	108 (64.7%)	34 (20.4%)	
Family income (n=153)				
<BRL 19,620.00	49 (32%)	39 (25.5%)	10 (6.5%)	0.066**
BRL 19,621.00 to BRL 32,700.00	36 (23.5%)	32 (20.9%)	4 (2.6%)	
BRL 32,701.00 to BRL 58,860.00	36 (23.5%)	26 (17%)	10 (6.5%)	
>BRL 58,860.00	32 (20.9%)	20 (13.1%)	12 (7.8%)	
Parents’ combined level of education (n=167)				
Some High School	27 (16.2%)	21 (12.6%)	6 (3.6%)	0.406**
High School	84 (50.3%)	65 (38.9%)	19 (11.4%)	
Higher Education	56 (33.5%)	38 (22.8%)	18 (10.8%)	
Mother’s BMI (n=160)	26.05 (4.96)	25.81 (4.80)	26.77 (5.38)	0.284*
Father’s BMI (n=141)	27.08 (3.70)	27.25 (4.01)	26.60 (2.57)	0.364*

Values presented as means (standard deviation) for the quantitative variables and n(%) for the qualitative variables; BMI: body mass index.

*p-value of the Student’s *t-*test; **p-value of the Chi-square test; BRL: Brazilian currency.

Consuming more whole milk, not having a television in the bedroom, and taking <2 physical education classes/week during childhood were significantly associated with the presence of asthma during adolescence. Being a girl and consuming more whole milk were associated with the presence of rhinitis during adolescence (Table 5).

**Table 5:** Logistic regression models to verify the influence of independent variables on the diagnosis of asthma and rhinitis.

	ß	Odds Ratio	95%CI	p-value
ASTHMA				
Categorical BMI (n=168)				
Underweight	1.648	5.14	0.67-39.62	0.116
Normal weight (Ref.)				
Overweight	-1.848	0.16	0.20-1.27	0.084
Obese	0.251	1.29	0.51-3.25	0.596
Fruit consumption (n=167)	0.188	1.21	0.94-1.56	0.149
Whole milk consumption (n=167)	0.216	1.24	1.03-1.49	**0.021**
Transportation to school (n=167)				
Active (Ref.)				
Passive	0.611	1.84	0.73-4.67	0.198
Television in the bedroom (n=165)				
Yes	-1.219	0.29	0.12-0.71	**0.007**
No (Ref.)				
Physical education classes (n=167)				
<2 classes/week (Ref.)				**0.018**
≥2 classes/week	-1.176	0.31	0.12-0.82	
Family income (n=153)				
>BRL 19,620.00	1.074	2.93	0.58-14.78	0.194
BRL 19,621.00 to BRL 32,700.00	1.099	3.00	0.56-16.07	0.200
BRL 32,701.00 to BRL 58,860.00	1.099	3.00	0.56-16.07	0.200
>BRL 58,860.00 (Ref.)				
RHINITIS				
Sex (n=168)				
Boys (Ref.)				
Girls	0.897	2.45	1.21-4.99	**0.013**
MPA (n=155)	-0.019	0.98	0.96-1.01	0.148
MVPA (n=155)	-0.012	0.99	0.97-1.00	0.110
VPA (n=155)	-0.028	0.97	0.94-1.01	0.109
Whole milk consumption (n=167)	0.193	1.21	1.05-1.41	**0.011**
Fast food consumption (n=167)	0.182	1.19	0.96-1.49	0.106
Transportation to school (n=167)				
Active (Ref.)				
Passive	0.624	1.87	0.88-3.97	0.106

Model developed based on variables that presented p <0.20; 95%CI: 95% confidence interval; Ref: reference; BMI: body mass index; BRL: Brazilian currency; MPA: moderate physical activity; MVPA: moderate-to-vigorous physical activity; VPA: vigorous physical activity.

## DISCUSSION

Several studies conducted in Brazil and worldwide have investigated lifestyle factors associated with the risk of asthma and rhinitis and the protection against these diseases.^7^ ^,^ ^8^ Genetic factors have been studied, but the great heterogeneity of the Brazilian population makes any conclusion difficult.^7^ Our study investigated several factors in childhood lifestyle that could be associated with the diagnosis of asthma or rhinitis in adolescence. Consuming more whole milk, not having a television in the bedroom, and attending <2 physical education classes/week at school presented higher risks for developing asthma; likewise, being a girl and having higher consumption of whole milk were risk factors for the presence of rhinitis.

In this study, the prevalence of asthma accounted for 15.5%, and the prevalence of rhinitis was 25.6%. These values were lower than those found by Barreto et al.,^4^ based on data from 109,104 children participating in the PeNSE survey, in which 23.2% of students in the 9^th^ grade of elementary school were identified with asthma, but are close to those observed by other national^6^ and Latin American^4^ studies whose authors followed the ISAAC protocol.

Significant differences were observed in categorical BMI between groups with and without asthma; however, the nutritional status was not associated with the development of the disease. A study that monitored 617 children from childhood (10-12 years old) to late childhood (12-13 years old) also found no association between BMI and the presence of asthma and rhinitis in late childhood.^10^ Another prospective study that investigated the relation between BMI in childhood (7-13 years old) and the diagnosis of asthma in adulthood (20-45 years old) found an association, though non-linear, according to which the high BMI increased the chances of having asthma in girls/women and low BMI in boys/men.^9^ Despite the aforementioned findings, a systematic review of six prospective cohort studies, which investigated the association between overweight or obesity (defined according to BMI) and the diagnosis of asthma, concluded that overweight children, and especially obese children, were at greater risk of having asthma diagnosed later than children with normal weight.^24^ Even with inconclusive results, we understand that the possible relationships between asthma and obesity are mainly due to the fact that both are systemic inflammatory diseases, and the inflammation caused by obesity can also affect the airways and accentuate the asthma diagnosis.^25^

We found that increased consumption of whole milk during childhood was significantly associated with an increased risk of developing asthma and rhinitis during adolescence. Conversely, a study investigated the effects of cow’s milk consumption on asthma and documented an inverse relationship between its consumption and prevalence of asthma, regardless of the fat content present in milk.^26^ It is noteworthy that, in this study, we did not detect differences in the skimmed milk consumption between the groups and neither an association with asthma or rhinitis, which suggests a concern about whole milk consumption only. Whole milk, unlike skimmed milk, is rich in lipids, saturated fatty acids, and myristic and palmitic acids, and these compounds have been previously associated with the risk of asthma in a study on Spanish schoolchildren (8-13 years old), which can corroborate our findings.^27^ Another controversial fact discussed by some authors is the association between cow’s milk consumption and the development of wheezing.^28^

Our hypothesis was that PA and SB could be associated with respiratory diseases. Despite the trend of PA being inversely associated with the development of rhinitis and the use of passive transportation increasing the risk of the disease, the values were not statistically significant. However, taking ≥2 physical education classes/week determined 70% protection for the development of asthma in relation to those children who attended it <2 times/week. These data reinforce the importance of physical education classes and PA during the week in schools and also the students’ participation. Physical education classes are excellent tools to fight overweight and obesity, in addition to being an important means to increase the level of PA.^29^ Although associations between asthma and nutritional status, as well as PA, were not observed, PA is paramount to help reducing body fat, thus preventing, in addition to obesity and overweight, inflammation in the airways present in asthma and rhinitis.

In addition to the indirect benefits previously reported, it is worth highlighting the direct benefits of physical exercise to the pathophysiology of these diseases such as improving physical fitness, strengthening the respiratory muscles, and reducing episodes of induced asthma and dyspnea. Such effects would positively impact the quality of life of those affected.^2^ ^,^ ^3^

Although having a television in the bedroom proved to be a protective factor against asthma, screen time did not differ between groups. The authors understand that such finding would be more relevant if associations with SB and the total screen time had also been found, which was analyzed both as continuous and categorical in nature.

It is noteworthy that some behaviors and habits acquired in childhood tend to remain in adolescence and adulthood and are difficult to modify.^30^ Although there was no reassessment of the habits and behaviors of children after the five years of the study, the authors believe they may have been maintained by most of the study participants.

The authors understand that the present study has some limitations: the sample does not allow extrapolation of data for Brazilian students; causality must be carefully considered, taking into account that we did not present whether such behaviors in childhood remained in adolescence, that is, after the five years of the study; in addition, only the asthma module of the ISAAC questionnaire was validated for application via telephone, unlike the rhinitis questionnaire.^16^ However, the prevalence of the disease was very similar to that found by ISAAC.^6^ Nevertheless, some points should be highlighted:

- The vast number of variables related to the lifestyle of both the child and the parents and the environment;
- PA and SB were measured using objective measures, which are still rare in Brazil;
- The quality and the guarantee of data collection procedures that followed a high rigor for standards and training.^12^

Understanding lifestyle factors is paramount to strategies aimed at fighting and preventing respiratory diseases. Further studies should be carried out in other Brazilian regions, considering that Brazil has a large population heterogeneity in its territory. Longitudinal studies are also required to observe such a causality.

It can be concluded that the diagnosis of asthma in adolescence was related to the higher consumption of whole milk, the absence of television in the bedroom, and the smaller quantity of physical education classes per week. Regarding rhinitis, factors that presented the highest risk were being a girl and consuming whole milk. Public policies must be directed toward a healthy lifestyle and the prevention of respiratory diseases both in childhood and in other stages of life.
